# Jasmonoyl-L-Tryptophan Disrupts IAA Activity through the AUX1 Auxin Permease

**DOI:** 10.3389/fpls.2017.00736

**Published:** 2017-05-08

**Authors:** Paul Staswick, Martha Rowe, Edgar P. Spalding, Bessie L. Splitt

**Affiliations:** ^1^Department of Agronomy and Horticulture, University of Nebraska–Lincoln, LincolnNE, USA; ^2^Department of Botany, University of Wisconsin–Madison, MadisonWI, USA

**Keywords:** tryptophan, conjugate, jasmonic acid, auxin, indole-3-acetic acid, phenylacetic acid, AUX1, gravitropism

## Abstract

Amide-linked conjugates between tryptophan (Trp) and jasmonic (JA) or indole-3-acetic (IAA) acids interfered with gravitropism and other auxin-dependent activities in Arabidopsis, but the mechanism was unclear. To identify structural features necessary for activity several additional Trp conjugates were synthesized. The phenylacetic acid (PAA) conjugate was active, while several others were not. Common features of active conjugates is that they have ring structures that are linked to Trp through an acetic acid side chain, while longer or shorter linkages are inactive or less active. A dominant mutant, called *tryptophan conjugate response1-D* that is insensitive to JA-Trp, but still sensitive to other active conjugates, was identified and the defect was found to be a substitution of Asn for Asp^456^ in the C-terminal domain of the IAA cellular permease AUX1. Mutant seedling primary root growth in the absence of added conjugate was 15% less than WT, but otherwise plant phenotype appeared normal. These results suggest that JA-Trp disrupts AUX1 activity, but that endogenous JA-Trp has only a minor role in regulating plant growth. In contrast with IAA- and JA-Trp, which are present at <2 pmole g^-1^ FW, PAA-Trp was found at about 30 pmole g^-1^ FW. The latter, or other undiscovered Trp conjugates, may still have important endogenous roles, possibly helping to coordinate other pathways with auxin response.

## Introduction

Indole-3-acetic acid (IAA) is a potent auxin hormonal signal that is tightly regulated to properly control plant growth and development. Regulation occurs at multiple levels including IAA biosynthesis, catabolism, and hormone inactivation by conjugation to amino acids and sugars (Ljung et al., 2002; Leyser, 2006). Additionally, IAA level in specific cells and tissues is controlled by regulating its transport, which is generally basipetal. In Arabidopsis polar transport is mediated by members of the plasma membrane-localized AUX1/LAX transporter family, which directs IAA into cells, while members of the PIN and ABCB protein families promote auxin export (Scheres and Xu, 2006; Spalding, 2013; Adamowski and Friml, 2015). Mutations in some of these transporters can markedly compromise plant development, confirming the critical role of IAA transport. For example, PIN efflux transporters help direct inflorescence development, lateral root formation, leaf veining and ovule formation, among others. Members of the AUX1/LAX influx transporters have similar functions, being necessary for root gravitropic response, proper lateral root development, root hair formation, vascular development, and phylotactic patterning (Swarup and Péret, 2012). IAA signaling is also controlled in complex ways downstream from its interaction with the auxin receptor TIR1 (Mockaitis and Estelle, 2008; Hagen, 2015; Strader and Zhao, 2016).

We previously described a unique activity for the amide-linked jasmonoyl-L-tryptophan (JA-Trp) conjugate in auxin response that was unrelated to the established role of jasmonoyl-L-isoleucine as the primary jasmonate signal (Staswick and Tiryaki, 2004; Adie et al., 2009; Staswick, 2009; Sheard et al., 2010). JA-Trp caused a striking agravitropic root growth response in Arabidopsis seedlings, inhibited auxin-stimulated lateral root development, and nearly eliminated seedling root inhibition caused by exogenously supplied IAA. Remarkably, IAA-Trp had similar activity and both conjugates were detected in Arabidopsis tissues, albeit at low levels, suggesting they may function as endogenous auxin regulators (Staswick, 2009). Activity required the TIR1 IAA receptor, but the conjugates did not inhibit IAA binding to TIR1 in *in vitro* pull-down assays.

In contrast with 1-N-naphthylphthalamic acid, localized application of JA-Trp or IAA-Trp to seedling shoot-root junctions did not inhibit lateral root development, indicating these conjugates are not IAA efflux inhibitors (Blakeslee et al., 2007; Staswick, 2009). On the other hand, IAA-Trp was still active in the *aux1-7* allele of the influx carrier, which might suggest AUX1 was unnecessary for conjugate activity. However, this experiment was confounded by the fact that *aux1-7* is a weak allele and its phenotype is the same as that produced by exogenous Trp conjugates.

These results suggested that coordination between IAA signaling and JA might be mediated through tryptophan conjugates. The purpose of this study was to further characterize the structural requirements for an active tryptophan conjugate, and to determine the molecular mechanism of Trp conjugate activity. Toward this end, we isolated a JA-Trp insensitive Arabidopsis mutant, called *tryptophan conjugate response1-D* (*tcor1-D*), and determined the defect was caused by an amino acid substitution in AUX1. This indicates that JA-Trp likely disrupts IAA cellular import mediated by AUX1.

## Materials and Methods

### Genotypes and Growth Conditions

All *Arabidopsis thaliana* plants were of the Col-0 ecotype. The *tcor1-D* mutant originated from a population of ethyl methanesulfonate mutagenized M2 seeds produced for another purpose in the *jar1-11* background, as described (Leyser and Furner, 1993). The mutant was backcrossed three times to wild type and restoration of the *jar1-11* allele to wild type was verified as outlined (Suza and Staswick, 2008). Seed for comparison of wild type and *tcor1-D* phenotypes in the absence of added Trp conjugates was obtained from three plants of each genotype grown together. The sextuple mutant *gh3.1,2,3,4,5,6* was previously described (Porco et al., 2016).

Plant growth conditions were as previously reported (Wei et al., 2015)_._ Seedling growth on MS agar medium was in an incubator at 22°C, 12 h day/night cycles, 150 to 200 μM s^-1^ m^-2^ and seedling root angles, length measurements and germination assays were done as previously described (Staswick et al., 1992; Staswick, 2009). Media supplementation with compounds are indicated in the figures. Trp conjugates showing minimal or no agravitropic response were tested at 50 μM.

### Trp Conjugate Synthesis and Quantification

Synthesis of new Trp conjugates and their purification was as previously detailed, with verification of the products by thin layer chromatography and gas chromatography/mass spectrometry (Staswick, 2009). The Trp-Trp dipeptide was purchased from GenScript (Piscataway, NJ, USA). Quantification of phenylacetic acid (PAA)-Trp was done as previously described for JA-Trp, using a stable isotope internal standard synthesized from PAA and ^13^C_6_-Trp (Staswick, 2009).

### DNA Extraction and Genetic Mapping

Genomic DNA for mapping and genotyping the mutant was prepared as previously reported (Staswick et al., 2002). F2 seeds from the cross of *tcor1-D* to Landsberg *erecta* (Ler) were grown 5 days on agar medium containing 50 μM DHJA-Trp, then plates were rotated 180°. Because the mutation was dominant we isolated homozygous individuals that were WT (not restored to the new gravity vector) after 24 to 48 h. After growth in soil leaf tissue was collected for DNA extraction. CAPS and SSLP markers used for bulk segregate analysis, and to identify flanking markers, are shown in Supplementary Table 1.

### Phenocopying the Mutant by Plant Transformation

Previously described methods were used for transformation of the *tcor1-D* cDNA to WT and *aux1-7* plants (Staswick and Tiryaki, 2004). Gene expression was driven by the *AUX1* wild type promoter encompassing 2514 bp upstream of the translation start site. Primers used to obtain this sequence from genomic DNA were GGATCCTCGTTGGGTAAAATCTGCAG and GGATCCAGATCTGAGAAATAAAACAGAGCG. The sequence “AGAAGC” 13 bp upstream from translation initiation was changed to a BamH1 recognition sequence to facilitate fusion to the *tcor1-D aux1* cDNA, obtained with primers GGATCCTAAAAAAATGTCGGAAGGAGTAGAAGCG and TCTAGATCAAAGACGGTGGTGTAAAGC. The CaMV 35S terminator region obtained from plasmid pRTL2 was added to the 3′ end of the full length *tcor1-D* coding sequence via Xba1 ends, and the entire transgene was incorporated into pZIP212 for transformation. Seeds from primary transformants were screened for the selectable marker (Kanamycin). Two transformants for each genotype that segregated 3:1 for Kan^R^: Kan^S^ were selected. From each of these homozygous transgenic lines (WT:5-2, WT:6-5, *aux*:6a, *aux*:13b) were developed, verified for the transgene and its expression, and used for phenotypic analysis. *AUX1* primers for the cDNA CAPS marker were AGAAAGTGATTGGGATGCATG and TCAAAGACGGTGGTGTAAAGC, and for the genomic CAPS marker were TTTCAGCCATTTCTTTATTTGG and CTTAGCACGCATTTAAAGGGG.

### Time-Lapse Image Analysis of Root Gravitropism

Seeds were sown on Petri plates containing a simple medium containing 1 mM KCl, 1 mM CaCl_2_, 5 mM MES, and 1% agar adjusted to pH 5.7 with Bis-Tris propane. Plates with seeds were stratified for 2 days at 4°C, then maintained vertically under continuous white fluorescent light at a photon fluence rate of approximately 50 μmol m^-2^ s^-1^. The temperature was approximately 20°C. After 4 days of growth, the plates were placed in front of computer-controlled CCD cameras equipped with close-focusing telephoto lenses as described by Durham Brooks et al. (2010). After a 1 h period of adaptation to the recording apparatus, the plates were rotated by 90° to initiate gravitropism. Images were automatically acquired every 2 min, and automatically analyzed as described by Durham Brooks et al. (2010).

## Results

### Structural Requirements for Trp Conjugate Activity

**FIGURE 1:**
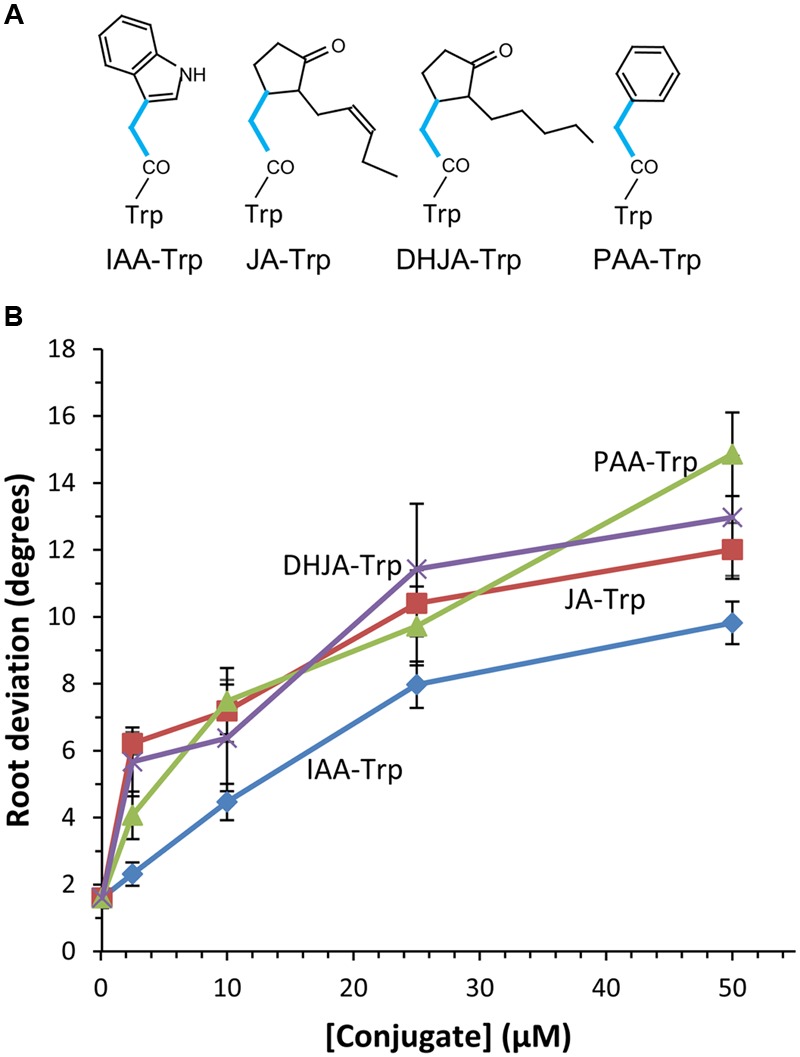
**Agravitropic activity of tryptophan (Trp) conjugates. (A)** The single carbon linkage between the ring structure of the acyl group and the amide linkage to Trp found in active conjugates is shown (blue lines). Abbreviations for the acids are noted in the text. **(B)** Agravitropic seedling root response to Trp conjugates after 6 days growth. Values are the means with SE (*n* = 30 seedlings). Significant differences from the control for each conjugate occurred at ≥1 μM for JA-Trp and DHJA-Trp, ≥2.5 μM for PAA-Trp and ≥10 μM for IAA-Trp (*p* < 0.01, *t*-test).

**FIGURE 2:**
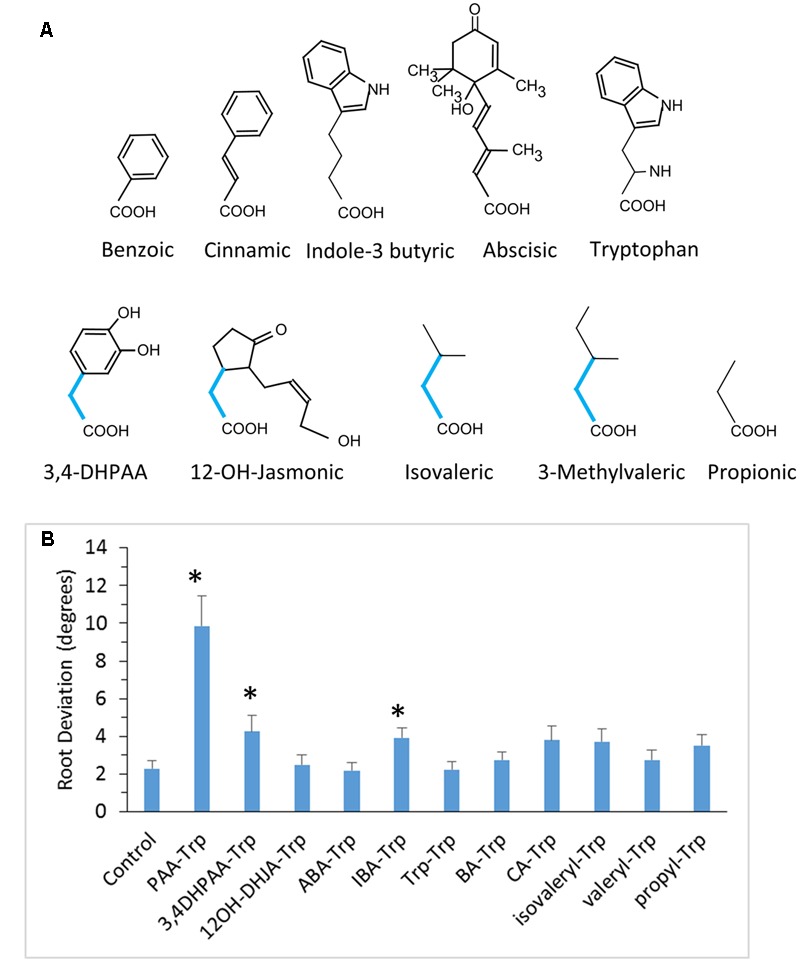
**Conjugates with little or no ability to disrupt root gravitropism. (A)** Structures of the relevant acids are shown. Bottom row shows acids with an acetic acid side chain that appears to be a required structural feature for active conjugates. **(B)** Root deviation for conjugates tested at 50 μM. Values are means with SE (*n* = 20 seedling roots). Asterisks indicate significant difference from control (*p* < 0.05, *t*-test).

Earlier experiments showed that IAA- and JA-Trp caused agravitropic root growth in Arabidopsis seedlings, while Trp conjugates of benzoic (BA) and cinnamic acids (CA) did not (Staswick, 2009). To further clarify the structural basis for activity we synthesized a number of Trp conjugates with other carboxylic acids, shown in **Figures 1A, 2A**. JA and IAA themselves are structurally diverse but they have in common an acetic acid side chain forming the linkage to Trp, whereas BA and CA are linked through one and three carbon carboxylic acid side chains, respectively. Thus we tested the PAA Trp conjugate, the two carbon side chain analog of BA, and found that it caused agravitropic growth similar to that of JA-Trp (**Figure 1B**). The dihydro analog of JA (DHJA) was also strongly active as a Trp conjugate. In contrast, indole-3-butyric acid, linked to Trp through a four carbon side chain was much less active than PAA-Trp, while the ABA conjugate and the Trp-Trp dipeptide were inactive (**Figure 2**). This suggests that although the structure of the carboxylic acid itself is not critical, spacing provided by the single carbon bridging the amide linkage to the ring structures of the carboxylic acids is important for activity.

Nevertheless, not all Trp conjugates coupled through an acetic acid moiety were active. The 3,4-dihydroxy derivative of PAA-Trp was weakly active and the 12-OH-jasmonic acid conjugate was inactive (**Figure 2**). Three conjugates with non-cyclic carboxylic acids were also inactive. We cannot rule out the possibility that inactive conjugates were not assimilated or were more readily catabolized than the active Trp conjugates. However, these results suggest that a bulky moiety spaced by a single carbon from the amide linkage is important for activity.

### Identification of a JA-Trp Insensitive Mutant

To elucidate the molecular mechanism of Trp conjugate activity an EMS mutagenized Arabidopsis M_2_ seedling population (representing about 13,000 M1 seeds) was screened for insensitive individuals. After 5 days growth on MS agar medium containing 50 μM DHJA-Trp, plates were rotated 180°. DHJA-Trp rather than JA-Trp was used for the screen because JA released from hydrolysis JA-Trp has root growth inhibiting activity, whereas DHJA does not. Putative insensitive seedlings were those that deviated minimally from the gravity vector in the first 5 days, and then reoriented their growth by 180° in response to the new gravity vector within 1–2 days, forming a pronounced hook.

**FIGURE 3:**
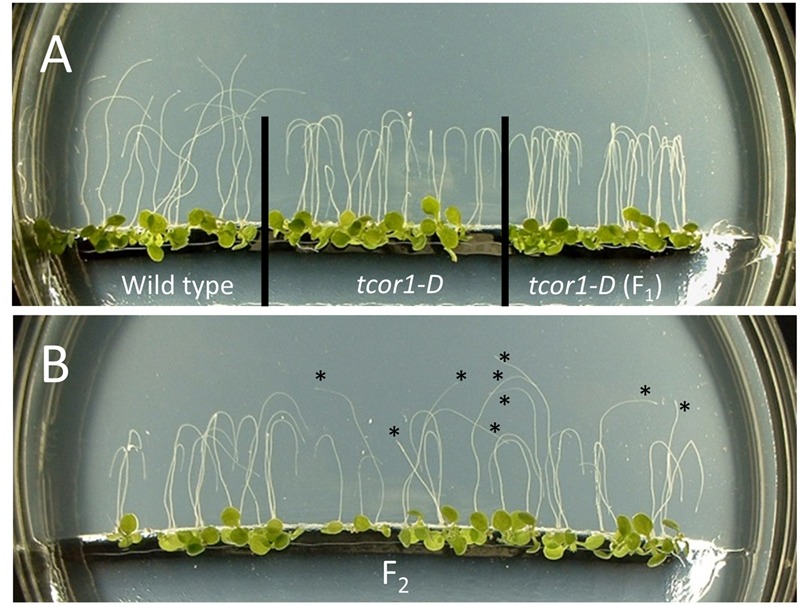
**Genetic characterization of DHJA-Trp insensitive mutant *tcor1-D*.** Seedlings were grown 4 days on MS agar plates with 40 μM DHJA-Trp, then rotated 180° for 36 h. **(A)** The impaired gravitropic response of wild type is evident, while roots of *tcor1-D* reoriented their growth in the direction of the new gravity vector (down in this image). The F_1_ was from a cross between wild type Col-0 (female) and homozygous *tcor1-D* (male). **(B)** F_2_ seedlings from a single F_1_ plant segregated 9:23 (sensitive: resistant; sensitive seedlings indicated with asterisks), consistent with a dominant mutation.

Only one putative mutant maintained a robust phenotype in subsequent tests. The original seedling was found to be heterozygous dominant for DHJA-Trp insensitivity and the mutant is called *tcor1-D*. **Figure 3A** shows the phenotypes of WT, homozygous *tcor1-D* and F_1_ seedlings from a cross between these two when grown under the same conditions as for the initial screen. F_2_ individuals from this cross segregated 9 sensitive: 23 resistant, consistent with a dominant mutation (**Figure 3B**).

### *tcor1-D* Is Specifically Insensitive to Jasmonate Conjugates

**FIGURE 4:**
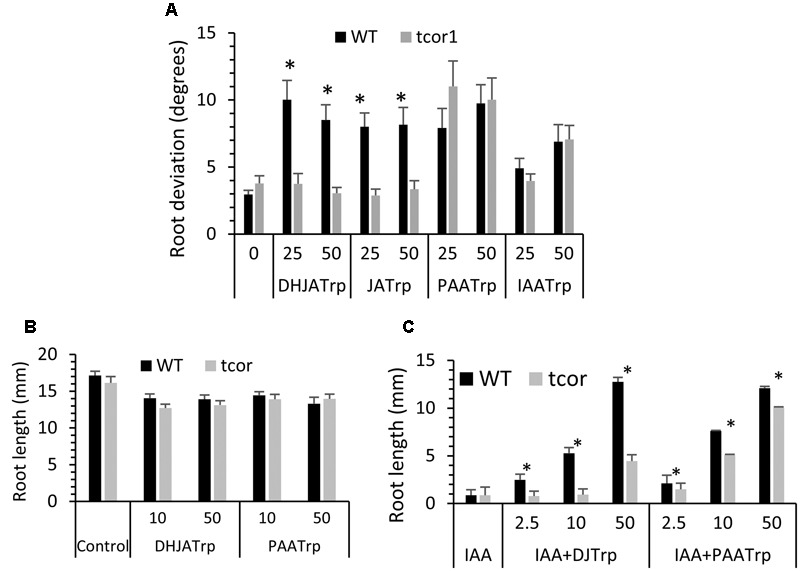
**Response of *tcor1-D* to Trp conjugates. (A)** Agravitropic response to conjugates at the indicated concentrations (μM). Growth conditions were the same as for **Figure 1**. **(B)** Seedling primary root length after 6 days growth on indicated conjugates (μM). **(C)** Same conditions as B but with 2 μM IAA included in medium for all tests. Values are means with SE, asterisks indicate significant difference between WT and mutant pairs (*t*-test, *p* < 0.01, *n* = 16–29).

We next tested *tcor1-D* for sensitivity to the active conjugates identified in **Figure 1** at 25 and 50 μM. **Figure 4A** shows there was no significant difference in mean root deviation from vertical compared to WT on control medium. The two jasmonate conjugates failed to affect gravitropic response of the mutant at either concentration, consistent with resistance to both conjugates. In contrast, *tcor1-D* had the same level of sensitivity to PAA-Trp and IAA-Trp as for WT, suggesting the insensitivity of the mutant was specific for the jasmonate conjugates.

Tryptophan conjugates were also previously shown to partially restore growth to seedling primary roots that were inhibited by exogenous IAA (Staswick, 2009). DHJA-Trp and PAA-Trp alone have only a small effect on root length after 6 days and there was no significant difference between WT and the mutant (**Figure 4B**). JA-Trp and IAA-Trp were not tested because conjugate hydrolysis releases small amounts of free JA and IAA, both of which are potent growth inhibitors that complicate interpretation of the results (Staswick, 2009). IAA at 2 μM strongly inhibits root growth in both genotypes, but DHJA-Trp and PAA-Trp at 50 μM restore WT growth to the level seen for these conjugates alone (compare WT in **Figure 4C** with 4B). In contrast the efficacy of DHJA-Trp was much lower in *tcor1-D* at all concentrations tested. The mutant was modestly insensitive to PAA-Trp in this assay, but not to the degree seen for DHJA-Trp. Together, these results show that the *tcor1-D* mutant has strong resistance to the jasmonate-Trp conjugates, but only weak resistance to PAA-Trp and IAA-Trp.

### JA-Trp Acts through AUX1

**FIGURE 5:**
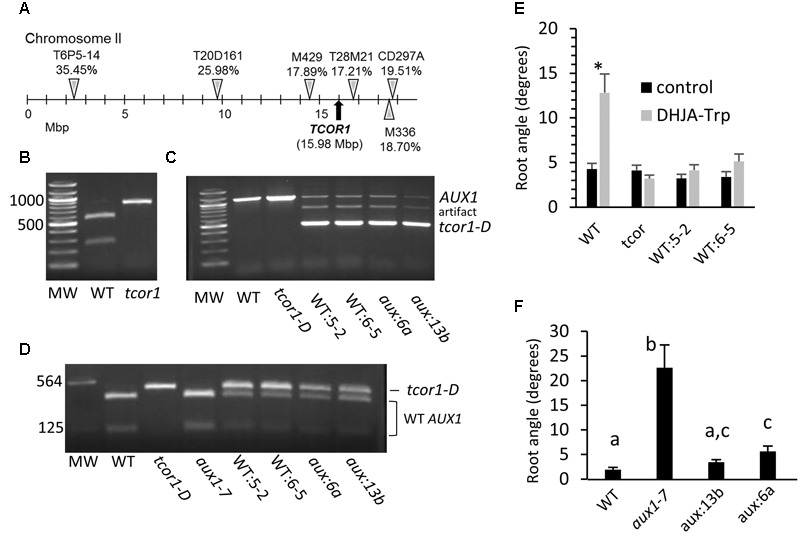
**Mapping of *tcor1-D* locus and transformation with the mutant *AUX1* cDNA. (A)** Details for the markers used are found in Supplementary Table 1. Percentages indicate proportion of recombinant chromosomes for individual markers seen among 110 to 123 F_2_ individuals each. **(B)** Genomic amplification products spanning the mutation site of the *AUX1* gene after cleavage with Sal1. **(C)** Uncleaved amplification products of *AUX1* genomic DNA from WT (WT:5-2, WT:6-5) and *aux1-7 (aux*:6a, *aux*13b) plants transformed with *tcor1-D* cDNA. **(D)** cDNA amplification products of indicated genotypes. Endogenous *AUX1* wild type cDNA is cleaved by Sal1, *tcor1-D* cDNA transgene is not. **(E)** Mean root angles with SE for WT seedlings transformed with *tcor1-D* cDNA on control and 25 μM DHJA-Trp medium. Significant difference between treatments for each genotype indicated by an asterisk (*t*-tests, *p* < 0.05, *n* = 14–36 seedlings). **(F)** Growth of *aux1-7* transformants on control medium. Means with same letters not significantly different (*t*-test, *p* ≤ 0.05, *n* = 17–21 seedlings).

To identify the gene affected in *tcor1-D* (Col-0 ecotype) molecular mapping was done in a segregating F2 population resulting from a cross to the Ler ecotype. Bulk segregate analysis indicated linkage on chromosome 2, and the locus was subsequently localized between flanking CAPS markers M429 and T28M21 near the right end of chromosome 2, shown in **Figure 5A**. Genes associated with auxin function in this chromosomal region include the IAA cellular importer *AUX1*. We sequenced the *AUX1* cDNA from the *tcor1-D* mutant to determine if any nucleotide changes were evident. The only difference from WT was a G to A substitution in the first nucleotide of the codon for Asp^456^, converting it to an Asn codon. Fortuitously, this eliminated a Sal1 site found in WT genomic DNA and cDNA. Genomic DNA analysis confirmed this mutation in *AUX1* from *tcor1-D* (**Figure 5B**).

To test whether the mutation in *AUX1* was responsible for conjugate insensitivity, the cDNA from *tcor1-D* was transformed into WT plants under the direction of about 2,500 bases of the WT *AUX1* promoter region. Presence of the transgene was verified by amplification of genomic DNA. The size of the endogenous gene fragment with these primers was 1126 bp, while the cDNA transgene was only 446 bp, due to lack of an intron present in the endogenous gene (**Figure 5C**). Homozygous transformants WT:5-2 and WT:6-5 had a single insertion site based on selectable marker segregation (not shown). Both the WT and cDNA transgene DNA bands were evident in these two transgenic lines (**Figure 5C**). (The intermediate-sized fragment was apparently a PCR artifact resulting from the transgene). Although the transgene copy number at each insertion site is unknown, the cDNA appears to be preferentially amplified, because the amount of total DNA was equal in each amplification reaction, yet the WT band was much weaker when the transgene was present. To verify that the *tcor1-D* cDNA transgene was expressed we amplified total cDNA across the region containing the polymorphic Sal1 site. After restriction enzyme digestion WT cDNA yielded fragments of 351 and 91 bp, while *tcor1-D* DNA produced a single fragment of 446 bp corresponding with the uncleaved amplification product (**Figure 5D**).

Analysis of the two WT transformants phenocopied with mutant *tcor1-D* cDNA showed that they acquired the *tcor1-D* insensitive phenotype for agravitropic response when grown on DHJA-Trp medium, while growth on control medium was not significantly different from WT seedlings (**Figure 5E**). The apparent wild phenotype of the *tcor1-D* mutant in the absence of exogenous Trp conjugates suggested that this *aux1* allele functions normally in auxin transport. We thus tested whether the *tcor1-D* transgene could restore normal gravitropic root growth in the agravitropic *aux1-7* allele. Genomic and cDNA amplification for the *aux*:6a and *aux*:13b transgenic plants gave similar results as for the WT transgenic plants (**Figures 5C,D**). (The *aux1-7* allele is indistinguishable from the WT allele in this PCR assay). Seedling root growth on control medium revealed that the two *aux1-7* transformants were restored to the same, or nearly the same, gravitropic response as for WT plants (**Figure 5F**). Collectively, these results demonstrate that the Asp^456^ conversion to Asn in *AUX1* is responsible for the insensitivity of *tcor1-D* to JA-Trp.

### *tcor1-D* Primary Root Has Reduced Growth Compared with WT

**FIGURE 6:**
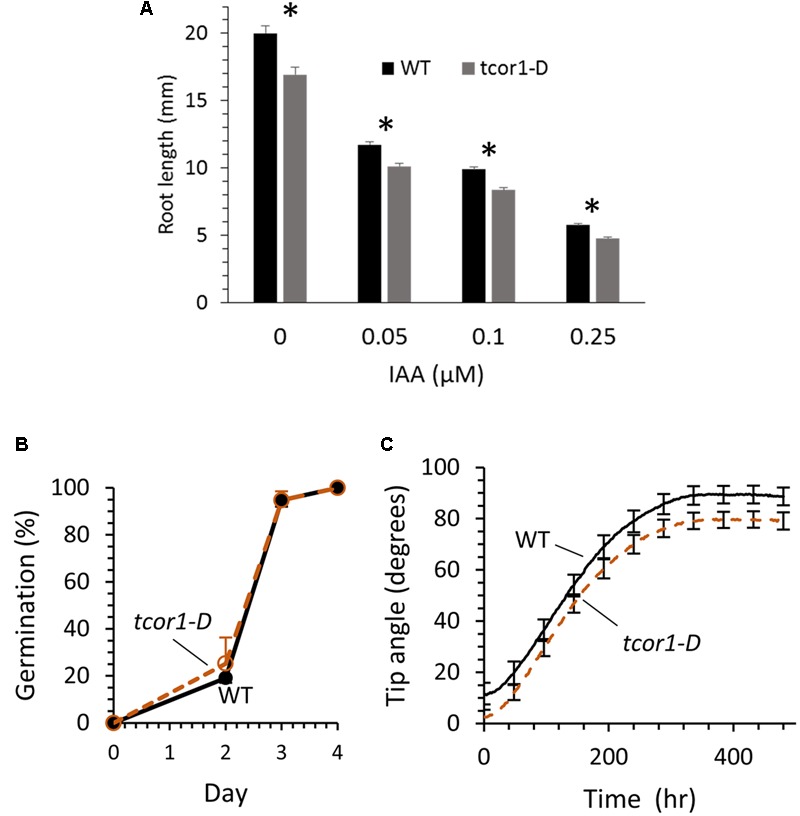
**Comparison of WT and *tcor1-D* growth. (A)** Mean root length for *tcor1-D* and WT seedlings grown 6 days in agar media with the concentration of IAA shown. Values are means with SE (*n* = 51–60 seedlings). Asterisks indicate significant difference between genotypes for each treatment (*p* < 0.01, *t*-test). **(B)** Germination rate for WT and *tcor1-D* seeds. Values are means for three experiments with SE (*n* = 28–35 seeds each experiment). Seeds were sown on MS medium and were counted as germinated when the emerged radical reached 1 mm in length. Growth was in a 16 h day/8 h night incubator at 21°C. **(C)** Combined gravitropic response for seedlings grown from seed obtained from three pairs of WT and *tcor1-D* plants. Tip angle measurements were taken every 2 h, SE of means shown only for every 24th time point.

Obvious growth or developmental defects were not readily apparent for *tcor1-D* when grown on control agar medium or in soil. To assess possibly more subtle effects of the *tcor1-D* mutation seedling primary root length was evaluated in agar medium with and without added IAA. To minimize confounding effects from differences in seed quality, seed was obtained from WT and *tcor1-D* plants grown together in a growth chamber. **Figure 6A** shows results after 6 days of growth. Roots were 15% shorter than WT for *tcor1-D* when grown on control medium, and similar differences were seen on media supplemented with 50–250 nM IAA. The two genotypes were tested for germination and all were essentially the same, indicating greater root length for WT was not due to earlier germination, but rather to slower growth for the mutant (**Figure 6B**).

Because Trp conjugates strikingly block root gravitropism we used a machine-vision method (Miller et al., 2007) to test whether the *tcor1-D* mutation affects the development of the gravitropic response in subtle ways in the absence of added conjugates. Root tip angles were measured automatically by software from images acquired at 2-min intervals. The responses of seedlings produced from three seed pools were combined to generate the results shown in **Figure 6C**. The only apparent difference between the *tcor1-D* and wild-type responses was a slightly greater initial tip angle for WT, apparently due to the observed modestly slower growth (**Figure 6A**). The first derivatives of the tip angle curves, or swing rates, (data not shown) were indistinguishable (Durham Brooks et al., 2010). Thus, despite slightly slower growth of *tcor1-D* roots the time courses and end-points of the gravitropism response were unaffected by the mutation.

### PAA-Trp Is Found at Higher Level Than Is JA-Trp and IAA-Trp

**FIGURE 7:**
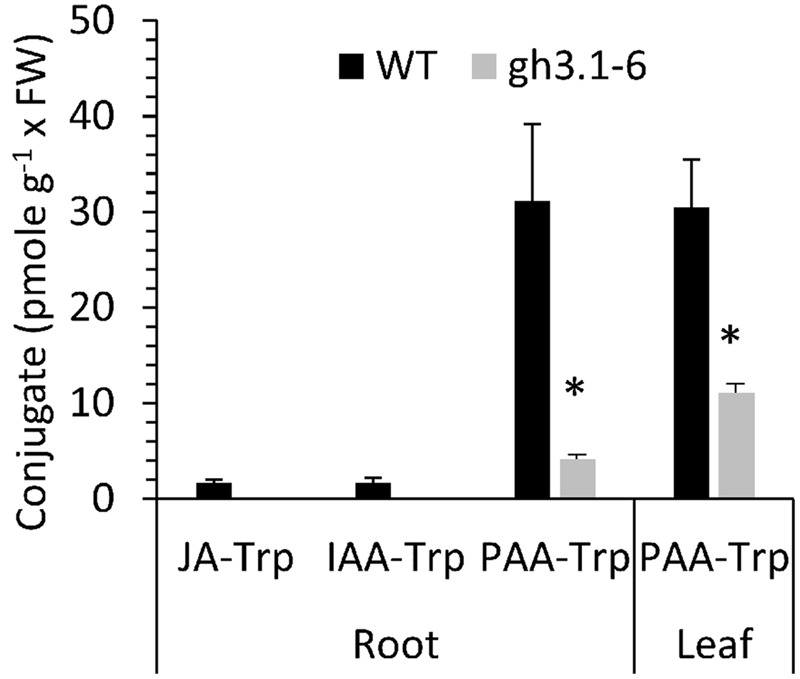
**Amount of PAA-Trp in Arabidopsis tissues.** Values are means for three independent biological replicates, with SE. Values for JA-Trp and IAA-Trp (Wild type only) determined previously (Staswick, 2009) are shown for comparison. Asterisks indicate significant difference between WT and sextuple mutant *gh3.1,2,3,4,5,6* (*p* < 0.05, *t*-test).

The presence of PAA-Trp was not previously reported in plants, but several GH3 conjugating enzymes are able to use PAA as an acyl substrate (Staswick et al., 2005). Although JA-Trp and IAA-Trp are only found at <2 pmole g^-1^ FW in Arabidopsis, **Figure 7** shows that PAA-Trp in seedling roots and leaves is about 17-fold higher. To determine whether GH3 enzymes synthesize PAA-Trp *in vivo* the sextuple mutant *gh3.1,2,3,4,5,6* was also examined (Porco et al., 2016). The amount of PAA-Trp was significantly lower in the mutant than in wild type, indicating at least some of these enzymes are partly responsible for synthesis of PAA-Trp.

## Discussion

**FIGURE 8:**
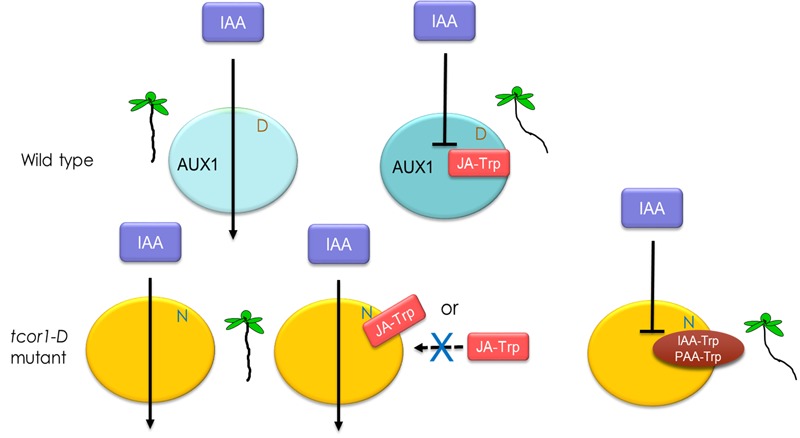
**Model for the action of Trp conjugates in IAA transport.** Turquoise ovals represent wild type AUX1 and yellow, the *tcor1-D* mutant version with the Asn for Asp substitution. Transport of IAA via AUX1 is indicated by arrows. Interaction of JA-Trp, IAA-Trp or PAA-Trp with AUX1 blocks IAA transport in wild type plants. In the mutant either JA-Trp doesn’t interact or does so in a way that does not interfere with IAA polar movement. The other conjugates are still effective at promoting an agravitropic root response, as indicated by the seedling representations.

We have established that JA-Trp acts through the AUX1 cellular auxin importer and it is likely that other Trp conjugates act similarly. The simplest hypothesis for the negative dominant nature of the *tcor1-D* mutant is that JA-Trp inhibits IAA transport in WT plants by binding to AUX1, but substitution of Asn for Asp^456^ in the mutant either eliminates conjugate binding or binding no longer interferes with IAA transport. This missense mutation has little impact on normal plant growth, indicating that the basic AUX1 transport mechanism remains functional in *tcor1-D*. A model illustrating this idea is shown in **Figure 8**. More complex models involving interaction with auxiliary factors are also possible, although no additional factors are required for AUX1 to transport IAA in *Xenopus* oocytes (Yang et al., 2006).

AUX1 is a permease with 11 transmembrane domains, the N- and C-terminal hydrophilic domains residing on the inner and outer plasma membrane faces, respectively (Swarup et al., 2004). The conditional *aux1-7* allele substitutes Asp for a conserved Gly at position 459 in the C-terminal tail and this allele is rescued from its agravitropic response by chromosaponin I (Rahman et al., 2001). Structure-function analysis suggested that the C-terminal domain may have a regulatory function, based on the conditional nature of the *aux1-7* defect and a possible regulatory role for the C-terminus of a related permease in animals (Sokolova et al., 2003; Swarup et al., 2004). Interestingly, the amino acid substitution conferring JA-Trp insensitivity in *tcor1-D* occurs just three residues upstream of the *aux1-7* substitution. If this region is indeed a regulatory domain, it is possible that JA-Trp does not directly block IAA transport, but instead acts as a negative regulator of AUX1 activity. This would be consistent with the apparent normal function of *tcor1-D* in the absence of added JA-Trp, with the caveat that slightly reduced primary root growth might result from insensitivity to endogenous JA-Trp.

The Asp equivalent to position 456 is conserved in all three of the closely related Arabidopsis LAX transporters. It is possible that one or more of these are endogenously regulated by JA-Trp, but none of them have major roles in gravitropic response (Swarup et al., 2004). The equivalent Asp is also conserved, though not invariant, in several other plant species. Data from a recent analysis of 44 AUX/LAX proteins from four legumes and three monocots reveals that 39 of these also have the conserved Asp, while one gene each from *Phaseolus vulgaris, Lotus japonicus, Sorghum bicolor*, and two from *Zea maize* encode the Asp to Asn conversion (Chai et al., 2016). So at least a few potentially JA-Trp insensitive AUX/LAX proteins occur naturally in plants.

The paradigm for hormonal activation by conjugation to an amino acid was established for JA-Ile, the primary active jasmonate signal (Staswick and Tiryaki, 2004; Adie et al., 2009; Sheard et al., 2010). Jasmonate and auxin signaling are connected at many levels, so it was attractive to think that conjugation of endogenous JA to a different amino acid (Trp) to regulate auxin activity could be another level of hormonal coordination. Although *tcor1-D* was strongly resistant to JA-Trp it had a near normal phenotype in the absence of exogenous JA-Trp, suggesting that any role for this particular conjugate is subtle, at least under the conditions tested here. The minor role for endogenous JA-Trp in response to exogenous IAA is supported by our previous finding that JA biosynthesis mutants, which should contain no JA-Trp, also had slightly reduced root growth in the presence of exogenous IAA (Staswick, 2009). We previously measured <2 pmole g^-1^ fresh weight for both JA-Trp and IAA-Trp in various WT Arabidopsis tissues, which is also consistent with a minor role.

Nevertheless, our results do not rule out the possibility of a significant role for other Trp conjugates in regulating AUX1 transport activity. Little or no insensitivity to IAA-Trp and PAA-Trp was found in *tcor1-D*, so they could be active participants in AUX1 regulation. We rescreened our mutagenized population for resistance to IAA-Trp and PAA-Trp but found no additional mutants to test this hypothesis. The substantially higher endogenous amounts of PAA-Trp in seedling tissues suggests it could play a more significant role than the other two endogenous conjugates. Although the sextuple *gh3* mutant that accumulates less PAA-Trp has auxin-related phenotypes, it is unclear whether this is due entirely to excess auxin resulting from compromised inactivating capacity, or if reduced PAA-Trp also contributes to the phenotype (Porco et al., 2016).

In summary, we solved the question of where JA-Trp acts to affect auxin responses. It is likely that other active Trp conjugates function similarly, and some may have important roles in controlling auxin transport.

## Author Contributions

Machine-based analysis of mutant phenotypes was planned, carried out and interpreted by ES and BS. All other aspects of the experimental work were done by PS and MR. PS did the project planning and manuscript writing.

## Conflict of Interest Statement

The authors declare that the research was conducted in the absence of any commercial or financial relationships that could be construed as a potential conflict of interest.
