# Supplementary material for: Jasmonoyl-L-Tryptophan Disrupts IAA Activity through the AUX1 Auxin Permease
Source: Front Plant Sci. 2017 May 8;8:736. doi: 10.3389/fpls.2017.00736 (PMC5420569; doi:10.3389/fpls.2017.00736)
Supplement: Supplementary file 1 [file Table_1.PDF]

Supplemental Table 1. Marker details for genomic mapping .

		Map Position	Primer	Primer	Annealing	MgCl2	Frag.Size	Restriction	Products (kbp)	
Chr.	Marker	(cM)	Direction	Sequence	Temp (°C)	(mM)	(kb)	Enzyme	Col	Ler
I	CAT3	29.91	F	CAGATGCAATGGCATCGTGGAG	55	1.5	0.973	Hinc II	0.973	0.790, 0.185
			R	CGGTGGTGCTCCAGTCTCCAAC						
I	UFO	49.6	F	AAG GCA TCA TGA CTG TGG TTT TTC	55	1.5	1.3	Taq I	0.983, 0.316	0.6, 0.383, 0.316
			R	GTG GCG GTT CAG ACG GAG AGG						
I	GAPB	92.6	F	TCT GAT CAG TTG CAG CTA TG	55	1.5	1.5	Bfa I	1.21, 0.21, 0.058	0.85, 0.366, 0.21, 0.058
			R	GGC ACT ATG TTC AGT GCT G						
I	ADH	117.5	F	GCG TGA CCA TCA AGA CTA AT	55	3.0	1.3	Xba I	1.291	1.097, 0.262
			R	AAA AAT GGC AAC ACT TTG AC						
II	PhyB	34.45	F	CAATCCTATGAAGAATGGCG	55	3.0	1.1	Xho I	1.1	0.7, 0.4
			R	ATAAACCATTAGCCCACGTG						
II	T20D161	9949768bp	F	CGTATTTGCTGATTCATGAGC	55	1.5	1.7	PSTI	1.4, 0.3kb	1.7kb
			R	ATGGTTTACACTTGACAGAGC						
II	m429	69.6/73.2???	F	TGG TAA CAT GTT GGC TCT ATA ATT G	55	3.0	0.3	ScrF I	0.316	0.216, 0.100
			R	GGC AGT TAT TAT GAA TGT CTG CAT G						
III	MYB4	4.73	F	CCAAATGACAACGACGTTATC	57	3.0	1.6	HaeIII	1.0, 0.6	1.2, 0.4
		46335bp	R	GCCGGGTTGAAGAAAGGGCC						
III	ALS	6	F	GGCAACACATGTTCTTGGTG	55-57	1.5	1.4	Hae III	0.952, 0.42	0.952, 0.22, 0.2
			R	ATCACAGGACAAGTCCCTCG						
III	g4711	38.1	F	CCTGTGAAAAACGACGTGCAGTTTC	55-57	1.5	1.5	Hind III	1.5	1.0, 0.5
			R	ACCAAATCTTCGTGGGGCTCAGCAG						
III	BGL1	87.1	F	TCT TCT CGG TCT ATT CTT CG	50	3.0	1.3	Rsa I	0.785, 0.34, 0.105	0.785, 0.485
			R	TTA TCA CCA TAA CGT CTC CC						
IV	F10N7H	5	F	CCTGCCCAATATGCCAAAGC	50	1.5	1.2	Hae II	1.0, 0.2	1.2
			R	GTGTATACATGCGTGTCAGC						
IV	GA1.1	17.7	F	CCGGAGAATCGTACGGTAC	55	3.0	1.196	BsaB I	0.707, 0.527	1.196
		1242594 bp	R	AAGCTTCGAACTCAAGGTTC						
IV	ch42	44	F	CATCTTCTTCTGCAATCTGGG	55	2.3	1.4	Cla I	0.75, 0.65	1.4
			R	CAGTGGATCTTTCCTCAGACG						
IV	DHS1	108.5	F	AGAGAGAATGAGAAATGGAGG	55	1.5	1.668	Dde I	1.491, 0.129, 0.048	1.62, 0.048
			R	CAAGTGACCTGAAGAGTATCG						
V	ASAI	18.35	F	CCTCTAGCCTGAATAACAGAAC	55	3.0	1.728	BclI	1.042;0.686 kb	0.686;0.553;0.489 kb
		1720605bp	R	CTTACTCCTGTTCTTGCTTAC						
V	PATI	6.1	F	GTATGAGAACATAGTAACCCCATG	55	1.5	1.9	Sph I	1.9	1.3, 0.6
		5956164 bp	R	GTCGACGTGGTGCGGTGGGTTG						
V	F13K20-T7	11021947 bp	F	TTTGTGCAATTTATTAGGGTAG	55	1.5	0.48	MSEI	2 fragments kb	3 fragments kb
			R	ATTTGCAGAAGTTGAAGTTGGTC						
V	PHYC.2	14007935 bp	F	CTACAGAATCGTCCTCAACG	55	1.5	2	PSTI	1.7;pieces<0.3 kb	0.8;0.7;
			R	CCTAATGGAGAATCATTCGG						several pieces <0.3 kb
V	DFR	63.4	F	AGA TCC TGA GGT GAG TTT TTC	50	1.5	1.1	BsaA I-C	0.609, 0.534	0.609, 0.318, 0.216
			R	TGT TAC ATG GCT TCA TAC CA						
V	LFY3	90.9	F	TAA CTT ATC GGG CTT CTG C	50-55	1.5	1.3	Rsa I	0.708, 0.236, 0.147,	0.855, 0.236, 0.126,
			R	GAC GGC GTC TAG AAG ATT C					0.126, 0.078…	0.078…
Additional Chromosome 2 markers										
II	T6P5-14	2.32	F	GAAAGGTTGTGAGAATGGCG	55	1.5	1.4	EcoR I	1.105, 0.319	1.4
			R	GGCAGAACTCATACCTCCAC						
II	T28M21	16.7	F	TCGTCTCGCCGTTCCTCTTC	55	1.5	3	Dra I	2.5, 0.5	3.0
			R	AGTCATCCAAGGCAACAAAG						
II	M336	18.5	F	CGTGAATGCTTTAGGAGTCC	55	1.5		SSLP	3.5	3.0
			R	TGGATTCGTTGCATGATGCG						
II	CD297A	18.8	F	CTCTTGGGAGAAGGCATAGG	55	1.5	2.8	Taq I	1.9, 0.9	1.1, 0.95, 0.75
			R	TGGAGAAATGGGTGTGAAGG						

CAPS and SSLP markers were identified through the TAIR portal (http://arabidopsis.org/portals/mutants/mapping.jsp). Additional markers were developed from published sequence polymorphisms between Columbia (Col) and Landsberg (Ler) ecotypes.
